# Reaching consensus on the definition of modifiable determinants of health: a Delphi study

**DOI:** 10.1136/bmjph-2025-004189

**Published:** 2026-02-16

**Authors:** Sebastian Stannard, Kim Alipio, Ann Berrington, Shantini Paranjothy, Rebecca B Hoyle, Rhiannon K Owen, Simon DS Fraser, Emilia Holland, Nisreen A Alwan

**Affiliations:** 1 NIHR Applied Research Collaboration Wessex, Southampton, UK; 2 School of Primary Care, Population Sciences and Medical Education, University of Southampton, Southampton, UK; 3 Department of Social Statistics and Demography, University of Southampton, Southampton, UK; 4 NHS Grampian Health Board, Aberdeen, UK; 5 School of Mathematical Sciences, University of Southampton, Southampton, UK; 6 Swansea University Medical School, Swansea University, Swansea, UK; 7 University Hospital Southampton NHS Foundation Trust, Southampton, UK

**Keywords:** Public Health, Epidemiology, Epidemiologic Methods

## Abstract

**Introduction:**

The term ‘modifiable risk factor’ and similar variations of the expression are common across health literature. Despite this, there is no universal definition for what would be modifiable when considering the factors that increase risk of ill health or enable good health and well-being. We conducted a Delphi study aiming to reach consensus among interdisciplinary experts on the definition and conceptualisation of what would be considered ‘modifiable’ as health determinants.

**Methods:**

The Delphi statements were based on initial criteria conceptualised by the research team and published in an opinion article. 103 experts from a range of interdisciplinary backgrounds were invited to participate in the Delphi. The statements were adjusted based on the results of the first round and circulated to participants in a second round.

**Results:**

33 experts completed the first round. 4 out of 10 statements achieved consensus (≥70%). 30/33 (90%) of experts completed the second round, and a further one out of three statements achieved consensus. Combining results from both rounds, we have reached this definition: ‘A modifiable health determinant must be potentially changeable through direct and/or indirect interventions at the individual or population levels, and it must be possible to quantify or describe such change in some way. Whether a health determinant is modifiable is context- and system-dependent (including the social, economic, political, commercial and environmental contexts); therefore, transparent consideration of a context-dependent definition is recommended in research design and reporting’.

**Conclusions:**

This study offers a consensus-based view on what can be considered ‘modifiable’. Having a common understanding of the term facilitates interdisciplinary collaboration in health research and translation of findings to policy and practice.

**WHAT IS ALREADY KNOWN ON THIS TOPIC**

- The term ‘modifiable’ is widely used to describe risk factors and determinants in health literature but lacks a universally accepted definition.

**WHAT THIS STUDY ADDS**

- This study aimed to reach expert consensus on defining and conceptualising modifiable health determinants.
- Our proposed definition is ‘A modifiable health determinant must be potentially changeable through direct and/or indirect interventions at the individual or population levels, and it must be possible to quantify or describe such change in some way. Whether a health determinant is modifiable is context- and system-dependent (including the social, economic, political, commercial and environmental contexts); therefore, transparent consideration of a context-dependent definition is recommended in research design and reporting’.

**HOW THIS STUDY MIGHT AFFECT RESEARCH, PRACTICE OR POLICY**

- Establishing a shared understanding of which health determinants are considered modifiable supports interdisciplinary collaboration and policy translation.
- We recommend that health researchers conduct a transparent consideration of what is labelled as ‘modifiable’ when designing and reporting studies.

## Introduction

 It is common to use the term ‘modifiable’ when referring to risk of disease or ill health, but there is no universally accepted definition of what constitutes a modifiable risk factor or determinant. This conceptual ambiguity may have implications for the consistency of research findings, the design of interventions and the formulation of public health policy.

The Oxford Learner’s Dictionary defines modification as the ‘act or process of changing something in order to improve it or make it more acceptable; a change that is made’.^1^ In health literature, the term ‘modifiable’ is frequently employed without a precise definition and often lacks clarification regarding the criteria by which a factor is deemed modifiable. In statistical discourse, however, it generally denotes a variable, factor or parameter that is subject to alteration or manipulation.^2^

In a previous comment piece,^3^ we argued that ‘in the absence of an agreed definition, transparent criteria to define what can be considered as modifiable determinants of health are desperately needed’.^3^ We proposed that what counts as modifiable depends on context and purpose, and we outlined five preliminary criteria for classification (online supplemental materials 1). To operationalise these criteria, we suggested guiding questions to help determine whether a factor can be considered modifiable, including:

1. Is it measurable?
2. Is it potentially changeable?
3. Are its causes modifiable in themselves?
4. Is it plausible as a cause?
5. Is there empirical evidence for its effect?

Finally, we called for transparent, context-sensitive definitions that include systemic determinants to better inform interventions and reduce health inequities globally. Explicitly defining what constitutes a modifiable risk factor can enhance the scope of public health planning and broaden the concept of modification to include the wider determinants of health, particularly for those conducting quantitative public health research. As discussed in our previous work, this approach is urgently required to effectively address health inequities both globally and within individual countries.^3^

Given the contextual and methodological complexities surrounding the concept of modifiability, fostering cross-disciplinary awareness and clarity is important. Therefore, building on this initial conceptualisation, we conducted a Delphi study to consult and achieve consensus among interdisciplinary experts on the definition and conceptualisation of what is meant by ‘modifiable risk factors’ in the context of health and disease outcomes.

## Methods

We used an adaptable Delphi technique.^4 5^ Unlike the classical Delphi approach, which typically begins with open-ended questions to generate ideas from the start of the process, the adaptable technique starts with a pre-selected list of items, in this case statements from our previous opinion piece.^3^ An adaptable Delphi technique often includes fewer rounds, the use of online tools or the incorporation of face-to-face discussions to clarify complex issues (although this was not explored in our Delphi). These adaptations make the method more practical and efficient while still preserving our core goal: achieving expert consensus through iterative feedback.^6^

### Recruitment

The inclusion criteria were: academic experts with experience of analysing modifiable risk factors or determinants across the life course for any health or disease-related outcome. Potential experts were identified by the research team using publicly available academic profiles and professional expertise. Invitations were extended to ensure representation across a broad range of disciplines relevant to health determinants research. This process was informed by the interdisciplinary nature of the research team. Experts were drawn from fields including public health, epidemiology and data science, social and population health, health sciences and oncology, medicine (including clinical medicine), global health and health economics. Authors of this article and colleagues in the wider National Institute for Health and Care Research (NIHR) Multidisciplinary Ecosystem to study Lifecourse Determinants and Prevention of Early-onset Burdensome Multimorbidity (MELD-B) project that triggered this work^7^ were excluded from participating. Potential experts were invited by email (online supplemental materials 2) to take part in the study and asked to consent (via a consent form) at the start of the first round of the Qualtrics (online supplemental materials 3). As part of the invitation email, potential experts were encouraged to read our published opinion piece that outlined why we were conducting the Delphi study.^3^ At the start of the first round survey, experts were asked if they would like to be named within the acknowledgement section of any published outputs or remain anonymous. We also asked experts to suggest others who they considered suitable to participate, who we then invited, provided they met the inclusion criteria after reviewing their publicly available academic profiles.

We used Qualtrics survey software^8^ to create and administer the survey. The survey was piloted among the research team before being published. We aimed to achieve a minimum sample size of 30 experts, given that a sample size of 20–30 has been found to provide a high level of replicability of between 64% and 77%.^9^ Potential participant experts had up to 15 days to complete each round of the survey and were sent three reminders per survey round.

### Round 1

Round 1 of the Delphi used a structured questionnaire included in online supplemental materials 3. The questionnaire comprised 10 statements relating to the concepts specified in our comment piece and illustrated in online supplemental materials 1.^3^ The experts were shown each candidate statement alongside a few exemplars to help stimulate thoughts and ideas. They were then asked to vote on a 5-point Likert scale. Potential responses were ‘strongly agree’, ‘agree’, ‘neither agree or disagree’, ‘disagree’ or ‘strongly disagree’ and ‘don’t know’. After each statement, there was a free-text box for experts to provide further comments. We also invited the experts to provide any further general comments in a free-text box at the end of the survey.

We conducted a manual review and synthesis of the free-text responses, systematically identifying and categorising recurring ideas into themes, which were subsequently defined and labelled.^10 11^ This was initially conducted by two of the research team (SS and NA) using Microsoft Excel, and then other authors (KA, AB, SP, RBH, RO, SDF) were invited to comment on the free-text response themes. Themes generated from the free-text responses were either incorporated into round 2 of the Delphi or were identified for discussion within the paper.

Although there is no universally defined consensus threshold,^12^ consensus was set a priori at 70% or more, as this struck a balance between achieving meaningful agreement and allowing for diversity of expert opinion, especially given our experts were from multiple disciplines. Therefore, if a statement achieved 70% or over consensus by agreement (either agree or strongly agree) or disagreement (either disagree or strongly disagree), it was excluded from the round 2 questionnaire. For those statements where consensus was not reached, the research team examined the free-text comments and either amended, removed or combined statements for the round 2 questionnaire.

### Round 2

Round 2 followed a similar structure to round 1, and the questionnaire for round 2 is included in online supplemental materials 4. Again, participants were asked to rate their agreement with each statement using a 5-point Likert scale, with an additional ‘don’t know’ option. Round 2 also included a ranking question where experts were asked to rank statements from most to least important as criteria for a factor to be modifiable. Each statement was followed by a free-text box for optional comments, and a final open-ended question invited further feedback. Again, free-text responses were reviewed and themes systematically generated and categorised, with two researchers (SS and NA) conducting the initial coding in Microsoft Excel, after which authors (KA, AB, SP, RBH, RO, SDF) reviewed and contributed to the interpretation. Consensus level remained at 70%.

### Round 3

A decision on whether we required a third round was made after reviewing and analysing results from rounds 1 and 2.

### Data collection and analysis

Baseline demographic data were collected during round 1 and presented as averages. These included age, gender, ethnicity, region of academic university or institute, disciplinary background and years of experience. Data were analysed using descriptive statistics within Excel.^13^

### Patient and public involvement statement

Patients or the public were not directly involved in the design, or conduct, or reporting, or dissemination plans of our research. However, this work and the opinion piece that it was based on were inspired by multiple discussions and considerations with public contributors and stakeholders during the MELD-B project in which we were attempting to define what is modifiable as lifecourse determinants of burdensome multiple long-term conditions.

## Results

### Participant expert characteristics

Out of the 103 experts invited to partake in the Delphi, 33 experts completed the first round of the survey (32% response rate). The second round was completed by 30/33 experts (90% response rate). The demographic characteristics of the experts are included in table 1.

**Table 1:** Demographic characteristics of expert participants included in the Delphi study

	Participant experts (n=33)
	N (%)
Gender	
Male	13 (39)
Female	20 (61)
Non-binary	0 (0)
Other	0 (0)
Prefer not to say	0 (0)
Age	
29 and under	0 (0)
30–39	2 (6)
40–49	14 (43)
50–59	9 (27)
60 years and over	8 (24)
Ethnicity	
Asian, Asian British or Asian Welsh	6 (18)
Mixed or other	2 (6)
White	25 (76)
Years of experience	
Less than 5 years	0 (0)
5–10 years	3 (9)
More than 10 years	30 (91)
Disciplinary background	
Public health	9 (28)
Epidemiology and data science	8 (25)
Social and population health	6 (19)
Health science and oncology	3 (9)
Clinical/medicine	3 (9)
Global health	2 (6)
Health economics	1 (3)
Location of affiliated university or research institute	
Within the UK	29 (88)
Outside of the UK	4 (12)

#### Round 1

The full results from round 1 are included in table 2. In round 1, the experts reached consensus on 4 out of 10 statements asked. These included:

A modifiable risk factor must be potentially changeable through direct and/or indirect interventions at the individual or population levels. (32/33 - 97% agreement)

Some of the criteria in the statements above must be fulfilled for a risk factor to be considered truly modifiable: Measurability, potential for change, modifiable cause(s), plausibility, and empirical evidence of effect. (27/33 - 82% agreement)

Some risk factors that were previously seen as ‘non-modifiable’, could be considered ‘modifiable’ under certain contexts. (24/33 - 73% agreement)

The extent to which a risk factor is modifiable depends on the disciplinary context and the social, economic, political, commercial and environmental context it is used in. (26/33 - 79% agreement)

**Table 2:** Statements for round 1 and 2, the distribution of results for each statement and the research decisions for amending statements for subsequent rounds

Round 1 statement (n=33)			Round 2 statement (n=30)		
Statement	Response	Decision on statement	Statement	Response	Decision on statement
Measurability: ‘For a risk factor to be modifiable, it must be measurable’	64% agree/strongly agree 33% disagree/strongly disagree	As consensus was not reached, we updated the wording for round 2 integrating experts’ comments from round 1	For a health determinant to be considered modifiable, it must be possible to quantify or describe in some way any potential change in it	90% agree/strongly agree 3% disagree/strongly disagree	Consensus reached after 2 rounds
Potential for change: ‘A modifiable risk factor must be potentially changeable through direct and/or indirect interventions at the individual or population levels’.	97% agree/strongly agree 3% disagree/strongly disagree	Consensus reached after round 1	–	–	–
Modifiable causes ‘The causes of the modifiable risk factors must be modifiable in themselves’.	42% agree/strongly agree 39% disagree/strongly disagree	As consensus was not reached we update the wording for round 2 integrating experts comments from round 1	The causes of the modifiable health determinants do not necessarily need to be modifiable in themselves.	63% agree/strongly agree 10% disagree/strongly disagree	No consensus after round 2 but unlikely to elicit different results if statement asked again
Plausibility: ‘For risk factors to be modifiable, they must be plausible as causes for the effect/ outcome’.	64% agree/strongly agree 18% disagree/strongly disagree	As consensus was not reached we update the wording for round 2, integrating experts comments from round 1	For a health determinant to be considered modifiable, there needs to be empirical evidence and/or theoretical understanding about its contribution to the causation of the outcome	67% agree/strongly agree 30% disagree/strongly disagree	No consensus after round 2, but unlikely to elicit different results if the statement is asked again
All of the criteria in the statements above must be fulfilled for a risk factor to be considered truly modifiable: Measurability, potential for change, modifiable cause(s), plausibility and empirical evidence of effect.	40% agree/strongly agree 52% disagree/strongly disagree	Giving the disagreement over whether all criteria were needed, we changed the question to a ranking statement in round 2 to identifying statements in order of importance	–	–	–
Some of the criteria in the statements above must be fulfilled for a risk factor to be considered truly modifiable: Measurability, potential for change, modifiable cause(s), plausibility and empirical evidence of effect.	81% agree/strongly agree 6% disagree/strongly disagree	Consensus reached after round 1	–	–	–
Some risk factors that were previously seen as ‘non-modifiable’, could be considered ‘modifiable’ under certain contexts	73% agree/strongly agree 15% disagree/strongly disagree	Consensus reached after round 1	–	–	–
Some risk factors should only be considered as partially modifiable, ie, the health risk posed by them can never be completely eliminated	67% agree/strongly agree 24% disagree/strongly disagree	It was agreed that this statement was covered by the other statements so it was removed for round 2	–	–	–
The extent to which a risk factor is modifiable depends on the disciplinary context and the social, economic, political, commercial and environmental context it is used in	79% agree/strongly agree 9% disagree/strongly disagree	Consensus reached after round 1	–	–	–
Do you think achieving consensus on the definition of modifiable risk factor is important?	45% yes 45% no	No further insight would be gained from asking this question again in round 2	–	–	–

For the six statements that did not reach consensus in round 1, the research team reviewed the accompanying free-text comments and proposed amendments, removals or combinations for round 2. These decisions are detailed in table 2. As a result, three statements were revised with updated wording to reflect expert feedback. Of the remaining three statements, two were removed either due to limited relevance, because no further insight would be gained from reissuing the statement, or because their content was sufficiently covered by other statements. One was reformulated as a ranking item to better capture expert preferences.

#### Round 1 free-text responses

In round 1, there was considerable disagreement over the use of the term ‘risk factors’, with one expert noting that: ‘the term ‘risk factor’ is still widely used in the health sciences but should be outlawed since it has no clear meaning*.* Does it mean ‘determinant’ (i.e. something that causes an outcome of interest) or ‘predictor’ (i.e. something that is associated with the probability of the outcome)?’. Another expert suggested that ‘risk factor terminology facilitates the continued mixing-up of modifiable exposures with the outcomes or consequences of exposures’. In response, we replaced ‘risk factors’ with ‘health determinants’ in round 2 to better reflect the intended meaning. For statements that had already achieved consensus, we updated the terminology without reissuing them, as the substantive content and interpretation remained unchanged. Further, we use the term health determinant instead of risk factor from this point onwards in the manuscript.

A further theme from round 1 was the complexity of defining and conceptualising what constitutes a modifiable health determinant. Several experts emphasised that modifiability does not necessarily depend on measurability. One expert noted that ‘a factor might be modifiable even if it is not measurable, or not easily measured,’ giving the example of ‘the amount of stress someone experiences, or how they experience stress, as it is a determinant for health and can be modified but not necessarily measured’*.* Another expert stated that ‘modifiability does not require quantifiability,’ while a further comment suggested that measurability should not be limited to quantitative terms, as ‘it might also be describable in qualitative ways,’. These reflections underscore the need for a more nuanced and inclusive understanding of modifiability.

Experts highlighted that the ability to eliminate a health determinant is highly context-dependent, with one expert noting the importance of distinguishing between what is ‘theoretically possible’ and what is ‘practically achievable’*.* For example, one expert stated that ‘some determinants may be theoretically eliminable, in reality, complete elimination is often unattainable’*.* It was also discussed that the term ‘modifiable’ typically implies that a ‘risk can be reduced rather than entirely removed, and does not necessitate total eradication’*.* Consequently, the use of ‘partially modifiable’ may be redundant, as ‘modifiable’ already encompasses a spectrum of possible reductions in risk. Ultimately, the extent to which a health determinant can be modified or eliminated varies significantly depending on the specific cause and context, and there may be strong grounds for believing something is modifiable even if it has not been demonstrated in the population.

#### Round 2

In round 2, three statements were presented to the expert. As shown in table 2, out of the three other statements from round 1 that did not reach consensus, two were removed and one was revised into a ranking item to better capture expert preferences. Additionally, some of the experts comments in round 1 were focused on the examples rather than the statements, and as such, we removed examples from round 2. Further, in round 1, the experts indicated that some of the statements for defining and conceptualising what is meant by a modifiable health determinant were more important than others. As such, in round 2, we included a new ranking question where experts were asked to rank the statements from most to least important.

Of the 33 experts who participated in round 1, 30 (90%) completed round 2. 2 male and 1 female participant did not complete the second round, resulting in a final gender distribution of 11 male and 19 female participants. The results from round 2 are included in table 2. Experts reached consensus on one out of three statements asked. This statement was:

For a health determinant to be considered modifiable it must be possible to quantify or describe in some way any potential change in it. (27/30 - 90% agreement)

The two statements that did not reach consensus in round 2 were:

The causes of the modifiable health determinants do not necessarily need to be modifiable in themselves. (19/30 - 63% agreement)

For a health determinant to be considered modifiable there needs to be empirical evidence and/or theoretical understanding about its contribution to the causation of the outcome. (20/30 - 67% agreement)

For these statements, the research team reviewed the accompanying free text. These statements approached the consensus threshold, with agreement levels of 63% and 67%, respectively. Given that the wording had already been revised following round 1, and the distribution of responses remained largely consistent across both rounds, the research team determined that a third round focused solely on these two items was unlikely to yield additional insights. Consequently, the Delphi process was concluded after two rounds.

**Figure 1:**
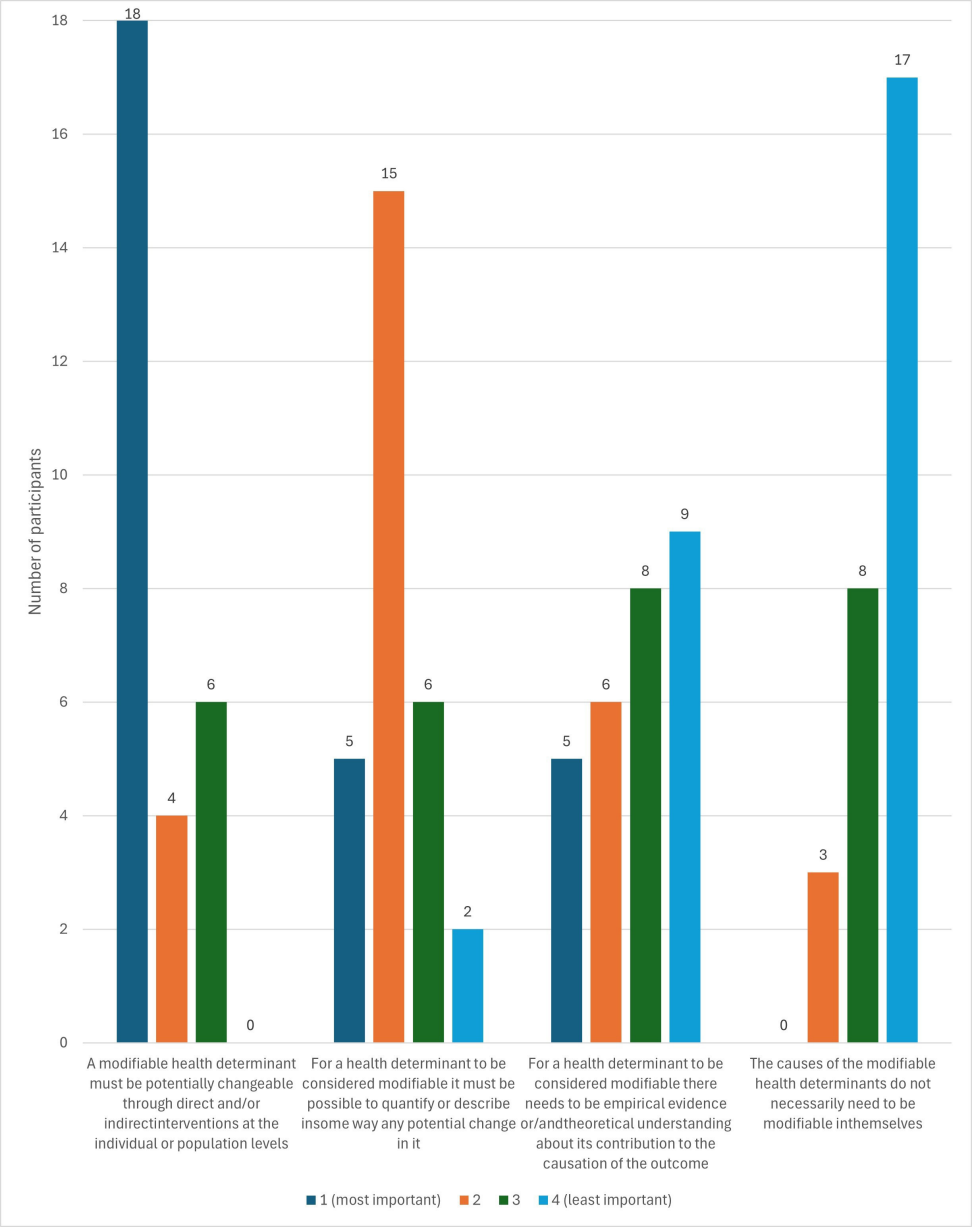
The distribution of ranking of statements from most important (1) to least important (4) (n=28).

As discussed above for round 2, we introduced a ranking statement where experts were asked to rank four statements in order of importance for defining a health determinant as modifiable, from most important (rank 1) to least important (rank 4). As shown in figure 1 the ranking of the statements was as follows:

(1) A modifiable health determinant must be potentially changeable through direct and/or indirect interventions at the individual or population levels. (18/28 - 64% of the experts ranked this statement 1^st^)

(2) For a health determinant to be considered modifiable it must be possible to quantify or describe in some way any potential change in it. (15/28- 54% of the experts ranked this statement 2^nd^)

(3) For a health determinant to be considered modifiable there needs to be empirical evidence or/and theoretical understanding about its contribution to the causation of the outcome. (8/28 - 29% of the experts ranked this statement 3^rd^ and 9/28 32% ranked it 4^th^)

(4) The causes of the modifiable health determinants do not necessarily need to be modifiable in themselves. (17/28 - 61% of the experts ranked this statement 4^th^)

#### Round 2 free-text responses

A key theme from the round 2 free-text responses was the complexity in measuring a modifiable determinant. A number of experts debated whether lack of measurability of a health determinant means that it is not, by definition, non-modifiable. Examples to support this argument, put forward by the experts, included ‘pollutants for which there are no accurate quantifiable tests’, and ‘climate change which is not easily quantified or even described but it is modifiable’.

## Discussion

This study presents a consensus-based perspective on defining modifiable health determinants of ill health and disease. In the first round of the Delphi process, with 33 expert participants, 4 out of 10 statements reached the predefined consensus threshold of 70% agreement. In the second round, with 30 expert participants, an additional one out of three statements achieved consensus and two further statements approached consensus, indicating a high level of agreement. By synthesising the statements that achieved consensus (from rounds 1 and 2) and the ranking of the most important statements (round 2), we propose the following definition of a modifiable health determinant:

A modifiable health determinant must be potentially changeable through direct and/or indirect interventions at the individual or population levels, and it must be possible to quantify or describe such change in some way. Whether a health determinant is modifiable is context- and system-dependent (including the social, economic, political, commercial and environmental contexts) therefore transparent consideration of a context-dependent definition is recommended in research design and reporting.

The Delphi process confirmed that core elements such as changeability and measurability are essential for any definition of a modifiable health determinant. However, experts emphasised that application must allow for adaptation across social, economic, political, commercial and environmental contexts. Therefore, our proposed definition sets out essential criteria while remaining adaptable to specific contexts, ensuring both clarity and relevance.

Although experts did not reach consensus on whether achieving a single definition of ‘modifiable’ is important, the Delphi process demonstrated that engaging experts in exploring and debating relevant statements serves a valuable role. This interaction promoted a critical and reflective approach to evaluating what constitutes a modifiable factor and helped assess whether a shared definition could be reached. Even without full agreement on its necessity, the process highlighted key conceptual considerations that can inform future research and practice.

We hope that through this Delphi process, we can offer a valuable framework for promoting critical reflection and standardising terminology. Establishing shared understanding could broaden the scope of modifiability to encompass social, environmental and structural determinants and encourage transparent approaches in health research design and reporting, which may also inform health policy and planning. For example, public health resources are limited, and a shared definition of what constitutes ‘modifiable’ can help transparent consideration during the research design and reporting stages around which determinants can realistically be changed through interventions, thereby adding clarity to evidence-based public health planning. Second, it improves consistency across research; without a clear definition point to start from, researchers will continue to label determinants loosely, making evidence translation challenging. Third, a shared definition supports equity-focused policies by broadening the scope of modifiability beyond individual behaviours to include systemic determinants that are often key drivers of health inequalities. Finally, a definition that incorporates context and flexibility can guide long-term planning. As experts noted, some determinants may become modifiable over time (eg, genetic factors through advances in gene editing), and anticipating such shifts helps future-proof public health strategies.

The Delphi process revealed that the understanding of what is considered modifiable in the context of health is not a fixed concept. There was consensus among experts that certain determinants previously viewed as ‘non-modifiable’ may, under specific circumstances, now be seen as modifiable due to evolving perceptions and scientific advances over time. For instance, several experts highlighted that aspects of genetics, once deemed non-modifiable, can now be altered through genetic engineering or gene editing. Moreover, the extent to which a health determinant is considered modifiable is highly dependent on the disciplinary lens through which it is examined, as well as the specific context, including population characteristics and broader social, economic, political, commercial and environmental conditions. This highlights the importance of flexibility and contextual awareness when defining and applying the concept of modifiability in health research and practice. The experts also consistently discussed across survey rounds the complexities in measuring a modifiable health determinant, with several experts describing that in situations where you cannot accurately measure a health determinant, it does not necessarily mean it is not modifiable. This further supports the need for some flexibility when considering a modifiable health determinant.

Given the contextual and methodological complexities highlighted through this study, achieving complete consensus on the definition of modifiable health determinants may not be possible. However, the experts agreed that the identification of important and relevant statements serves a valuable role in promoting a critical and reflective approach when evaluating what constitutes a modifiable factor. The statements identified in this research can act as overarching principles to help standardise terminology and encourage cross-disciplinary dialogue. Importantly, the discussions presented here underscore the need for clarity in the use of the term ‘modifiable’. Given the significance of context, we recommend that all authors explicitly define and justify their use of ‘modifiable’ within the scope of their own work, ideally drawing on the guiding statements presented in this paper to support their rationale. Further, while not specifically related to the term ‘modifiable’, recent literature supports the need for clearer and well-defined terminology to assist researchers, healthcare providers, policymakers, patients and family caregivers.^14^ The diversity of audiences and the broad scope of health-related research highlight the importance of using consistent and well-defined terminology to foster communication, promote collaboration and avoid misunderstandings.^14^

It was highlighted by many experts that the use of the term ‘risk factor’ facilitates the continued mixing-up of modifiable exposures with the outcomes or consequences of exposures, and despite its widespread use, the term has no clear meaning, and could refer to either a ‘determinant’ or ‘predictor’. Several experts suggested that the term ‘risk factor’ may no longer be fit for purpose and should potentially be avoided altogether in health-related research. Although this issue was not the primary focus of the present study, the persistent ambiguity surrounding the term ‘risk factor’ highlights the need for future research to achieve clarity on its meaning. A similar consensus-based approach could be used to explore whether agreement can be reached among experts on the appropriate use and interpretation of the term ‘risk factor’ across health disciplines.

Given the discussion from experts around the use of the terms ‘risk factors’ the decision to use the term ‘modifiable determinant’ rather than ‘modifiable risk factor’ reflected both expert consensus and conceptual considerations. As discussed, Delphi participants strongly recommended moving away from ‘risk factor’. While the broader framing of determinants aligns with contemporary public health approaches that emphasise upstream and systemic drivers of health, rather than focusing solely on risk reduction. We acknowledge that the term ‘modifiable determinant’ is not without ambiguity and may require further refinement; its flexibility could also be a strength as it supports integrated strategies for health improvement and policy development across multiple contexts. During the design of the second round, we considered including a Delphi question around the term ‘modifiable determinant’. However, we decided against this because it would have distracted from the primary aim of achieving consensus on defining modifiability. We believe this issue warrants a separate consensus exercise, given its conceptual complexity and potential implications for public health research. Therefore, although the term ‘modifiable determinant’ is increasingly used in the literature, its appropriateness might depend on context, and work is required to ensure clarity and consistency through another Delphi study.

This Delphi study was conducted with highly proficient experts from a range of health-related academic disciplines, ensuring a diverse array of perspectives and viewpoints. We achieved a strong response rate between rounds (90%), which maintained the continuity and consistency of expert input throughout the process. This high level of engagement enhanced the reliability of the consensus reached and contributes to the overall validity and credibility of the study. However, a limitation of the study was the relatively low number of international participants; only four experts were affiliated with academic or research institutions outside the UK. Nonetheless, within the UK, we achieved a broad geographical distribution of experts from across the country. However, our experts lacked diversity, with 76% identifying as white and 94% over the age of 40, and no participant identified as non-binary or other gender. This demographic imbalance may limit the range of perspectives represented, particularly those of early-career researchers and individuals from other ethnic groups. Finally, although this work was inspired by multiple discussions and considerations with public contributors, we acknowledge that not including public and patient contributors directly in the Delphi process is a limitation. We will endeavour to involve patients and the public in any future planned Delphi, for example, on the term risk factor.

## Conclusion

This study provides a definition for the term ‘modifiable’ which is widely used to describe health determinants while lacking clear and consistent criteria to define it. We achieve this through a consensus-based approach towards encouraging greater clarity and critical reflection in the application of such terms. The findings support a broader and more nuanced understanding of ‘modifiable’, extending its scope to include social, environmental and structural determinants of health. This expanded perspective is essential for promoting more integrated approaches to health research, policy and planning and represents an important step toward addressing global health inequities. Furthermore, this study highlights the need for similar consensus-driven efforts to clarify other commonly used but conceptually ambiguous terms in health research such as ‘risk factors’ and ‘health determinants’.

## Supplementary material

10.1136/bmjph-2025-004189 online supplemental file 1

## Data Availability

Data sharing not applicable as no datasets generated and/or analysed for this study.
