# Supplementary material for: Reaching consensus on the definition of modifiable determinants of health: a Delphi study
Source: BMJ Public Health. 2026 Feb 16;4(1):e004189. doi: 10.1136/bmjph-2025-004189 (PMC12911794; doi:10.1136/bmjph-2025-004189)
Supplement: online supplemental file 1 [file bmjph-4-1-s001.docx]

**Supplementary Materials**

**Supplementary Materials 1. Initial criteria to consider before classifying health determinants as ‘modifiable’ [3].**


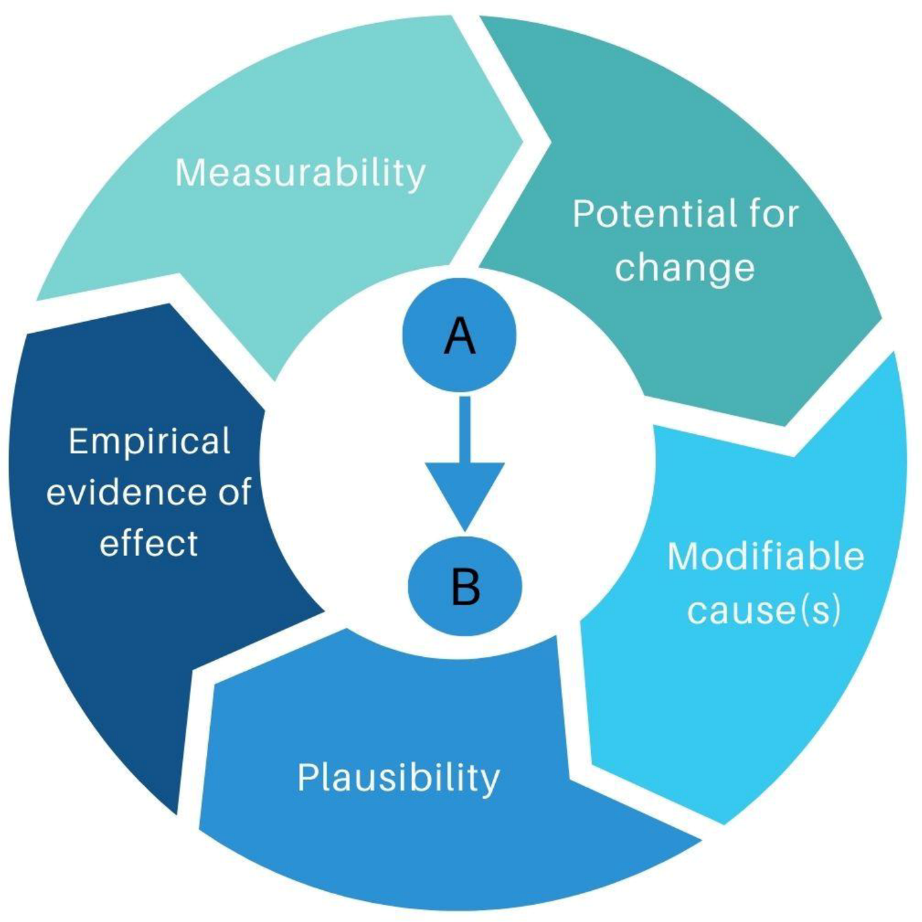


**Supplementary Materials 2: Invitation Email**

Dear X,

We are conducting a Delphi survey entitled: “Reaching interdisciplinary expert consensus on what is considered a modifiable risk factor for health: a Delphi study”(ERGO 99853) and we would like to invite you to participate. Your expertise would greatly contribute to the success of this study.

This Delphi study aims to consult and achieve consensus among interdisciplinary experts on a structured framework to define and conceptualise what is meant by ‘modifiable risk factors’ in the context of ill health and disease outcomes. The purpose of doing so is to facilitate public health planning through evidence-based interventions to prevent ill health. This is urgently needed if we want to tackle health inequities both on the global and within-country scales.

The modifiable risk factors DELPHI study follows on from the opinion piece: [‘Risk factors for ill health: How do we specify what is ‘modifiable’? (Alwan et al.).](https://eur03.safelinks.protection.outlook.com/?url=https%3A%2F%2Fjournals.plos.org%2Fglobalpublichealth%2Farticle%3Fid%3D10.1371%2Fjournal.pgph.0002887&data=05%7C02%7CS.J.Stannard%40soton.ac.uk%7C75ff61ea2dd04bd6fff308dd66100de9%7C4a5378f929f44d3ebe89669d03ada9d8%7C0%7C0%7C638778941309948060%7CUnknown%7CTWFpbGZsb3d8eyJFbXB0eU1hcGkiOnRydWUsIlYiOiIwLjAuMDAwMCIsIlAiOiJXaW4zMiIsIkFOIjoiTWFpbCIsIldUIjoyfQ%3D%3D%7C0%7C%7C%7C&sdata=UO2Nq2gnSIuGjc5Rhylj%2BPSwJRNOqmenM1bzgjvz8Js%3D&reserved=0)which recommended establishing consensus on the definition of modifiable determinants of health.

The survey is based on the conventional Delphi method. The process involves initially administering a questionnaire to a panel of experts. A follow-up questionnaire is crafted based on the responses from the first round, further refining and clarifying the consensus statements. The responses of each round of the Delphi are summarised and presented as aggregate findings before the start of the next round. These findings and any comments will not be credited to any participant[s] and hence will be anonymous.

You will be able to decide whether you want public acknowledgement of your role in the Delphi study. If you wish your contribution to be acknowledged, and consent to do so, you will be acknowledged by name in the published findings from the Delphi study.

Your Role: As a respected expert, your insights are invaluable. Your participation involves responding to a series of questions over a maximum of three rounds. The approximate time needed to complete this round is around 15-20 minutes.

Timeline: Participants will have a maximum of 15 days per round to complete the survey, from when the survey was first issued. Participants will be sent a maximum of three reminders per round to complete the survey.

How to Participate: If you're willing to take part, please review the attached Participant Information Sheet.  You can ask us any questions about the study via email. You can then access the survey link provided below to give your consent, and participate in the survey.

Please do not share this link with others, as it is intended solely for you as a personal invitation. If you would like to suggest other experts to take part in this activity, please email us with your suggestion on [S.J.Stannard@soton.ac.uk](mailto:S.J.Stannard@soton.ac.uk)

Follow this link to the Survey:

Take the survey

Or copy and paste the URL below into your internet browser:

https://southampton.qualtrics.com/jfe/form/SV_agjXYPVHvAxqgdg?Q_CHL=gl&Q_DL=EMD_snSfnfHgL0OPGRn_agjXYPVHvAxqgdg_CGC_HLXlG23t58sM9cx&_g_=g

Your expertise and contribution will be highly appreciated. Thank you for considering this invitation.

Thanking You,

Professor Nisreen Alwan, Dr Sebastian Stannard and Dr Kim Alipio

School of Primary Care, Population Sciences and Medical Education,  
Faculty of Medicine,  
University of Southampton,

**Supplementary Materials 3: Qualtrics Round 1 Survey**

Start of Block: Consent

**Study Title:** Reaching consensus on what is considered a modifiable risk factor: a Delphi study Thank you for your interest in this study. It is very important to us to conduct our studies in line with ethics principles, and this Consent Form asks you to confirm that you agree to take part in the above study. Please carefully consider the statements below and tick if you agree to participate in this research and understand what this will mean for you.

**Please tick the boxes below if you agree with the statements:**

|  | Tick (4) |
| --- | --- |
| I confirm that I read the Participant Information included in the invitation email that explains the study above and I understand what is expected of me. (1) |  |
| I was given the opportunity to consider the information, ask questions about the study, and all my questions have been answered to my satisfaction. (2) |  |
| I agree to take part in this study and understand that data collected during this research project will be used for the purpose of this study. (3) |  |
| I understand that my participation is voluntary and that I am free to withdraw from this study at any time without giving a reason. (4) |  |
| I understand that my quotes may be used in research reporting in anonymised format. (5) |  |
| I understand that if I withdraw from the study, it may not be possible to remove my data once my personal information is no longer linked to the study data. I understand that I can withdraw my data from the use in this study within 2 weeks following my participation. (6) |  |

End of Block: Consent

Start of Block: Introduction

Risk factors of ill health and disease are often referred to as being ‘modifiable’ or not, however there is no clear definition for this. This Delphi study aims to consult and achieve consensus among interdisciplinary experts on a structured framework to define and conceptualise what is meant by ‘modifiable risk factors’ in the context of ill health and disease outcomes. The purpose of doing so is to facilitate public health planning through evidence-based interventions to prevent ill health. This is urgently needed if we want to tackle health inequities both on the global and within-country scales. The modifiable risk factors DELPHI study follows on from the opinion piece: ‘**Risk factors for ill health: How do we specify what is ‘modifiable’? (Alwan et al.)** which recommended establishing consensus on the definition of modifiable determinants of health. Please note that for each statement below, we provide an example to illustrate the point. The example may have been drawn from the wider literature and if so this is signified by **underlined and bold text**, which is a hyperlink to the relevant paper. However, these examples are far from exhaustive, and respondents are free to think of their own examples and scenarios if this better helps them to form their opinion about each statement. For clarity, the statements on which we are asking your agreement or disagreement with are in italics, while the corresponding examples are below each statement. Under each of the statements below there is a free text box. Please use it provide any additional comments about the corresponding statement. At the end of this survey, there is also a free text box to use about any general thoughts you may have. The survey will take approximately 15 - 20 minute to complete.

| Page Break |  |
| --- | --- |

End of Block: Introduction

Start of Block: Demographics

Please state your professional title and name

________________________________________________________________

Please state your affiliation to either an academic or professional organisation

________________________________________________________________

Please state your age category:

- Under the age of 30 years (1)
- 30 - 39 years (2)
- 40 - 49 years (3)
- 50 - 59 years (4)
- 60 - 69 years (5)
- 70 years or above (6)
- Prefer not to say (7)

Please state your gender:

- Male (1)
- Female (2)
- Non-binary (3)
- Prefer not to say (4)
- Other (please specify in the box below): (5) __________________________________________________

Please state your ethnicity

- Asian, Asian British or Asian Welsh (1)
- Black, Black British, Black Welsh, Caribbean or African (2)
- Mixed or Multiple Ethnic Groups (3)
- White: English, Welsh, Scottish, Northern Irish, Irish Gypsy or Irish Traveller, Roma or Other White (4)
- Prefer Not to Say (5)
- Other Ethnic Group (please specify in the box below): (6) __________________________________________________

Please choose the category that corresponds to your years of experience in your area of expertise.

- Less than 5 years (1)
- 5 - 10 years (2)
- More than 10 years (3)

Do you wish to be identified and individually acknowledged by name in the report of the DELPHI findings?

- Yes (1)
- No (2)

End of Block: Demographics

Start of Block: Please indicate the extent to which you agree with the following statements:

Q1 **1) Measurability:** *‘For a risk factor to be modifiable, it must be measurable’* Examples: **Smoking** can be measured by its absence, dose, type. **Physical activity** can be measured by the length, intensity and type. **Alcohol use** can be measured by units per week, or questionnaires (e.g. Alcohol Use Identification Test, or AUDIT)

- Strongly Agree (5)
- Agree (4)
- Neither Agree nor Disagree (3)
- Disagree (2)
- Strongly Disagree (1)
- Don't Know (6)

Please feel free to add additional comments below:

________________________________________________________________

| Page Break |  |
| --- | --- |

Q2 **2) Potential for change:** *‘A modifiable risk factor must be potentially changeable through direct and/or indirect interventions at the individual or population levels’.*   Examples: Direct interventions **Smoking:** smoking cessation programmes at the individual level. Taxation at the population level. **Alcohol use:** counselling and talking therapies at the individual level. Reducing alcohol outlet density and minimum unit pricing at the population level. **Teenage pregnancy:** access to contraception at the individual level. Comprehensive sex education at the population level. Indirect interventions  **Smoking:** reducing workplace and occupational stress at the individual level. **Alcohol use:** rent assistance influencing behaviours at the individual level. **Teenage pregnancy:** curriculum changes to promote self-esteem and inclusive ethos at school may modify maternal age at conception at the population level.

- Strongly Agree (5)
- Agree (4)
- Neither Agree nor Disagree (9)
- Disagree (8)
- Strongly Disagree (7)
- Don't Know (6)

Please feel free to add additional comments below:

________________________________________________________________

| Page Break |  |
| --- | --- |

Q3 **3) Modifiable cause(s):** *‘The causes of the modifiable risk factors must be modifiable in themselves.’* Examples: **Type of household occupation and household income:** can be considered ‘parent’ causes to living in a deprived area which is a risk factor of mortality in people infected with COVID-19. **Social isolation and lack of transportation:** can be considered ‘parent’ causes to undernutrition, which is a cause of stunted growth in children. **Poor sleep quality:** can be considered a ‘parent’ cause to physical inactivity, which is a cause of increased depressive symptoms.

- Strongly Agree (4)
- Agree (6)
- Neither Agree nor Disagree (3)
- Disagree (2)
- Strongly Disagree (1)
- Don't Know (5)

Please feel free to add additional comments below:

________________________________________________________________

| Page Break |  |
| --- | --- |

Q4 **4) Plausibility:** *‘For risk factors to be modifiable, they must be plausible as causes for the effect/ outcome.‘* Examples: **Certain types of frontline occupations** such as healthcare, social care or teaching can plausibly lead to increased exposure to SARSCoV2. The effect of a smoking cessation programme on cardiovascular disease risk. The effect of **infant sleeping position** on the risk of sudden infant death syndrome (SIDS).

- Strongly Agree (5)
- Agree (4)
- Neither Agree nor Disagree (9)
- Disagree (8)
- Strongly Disagree (7)
- Don't know (6)

Please feel free to add additional comments below:

________________________________________________________________

| Page Break |  |
| --- | --- |

Q5 **5)** *‘****All of the criteria*** *in the statements above must be fulfilled for a risk factor to be considered truly modifiable: Measurability, potential for change, modifiable cause(s), plausibility, and empirical evidence of effect.’*

- Strongly Agree (5)
- Agree (4)
- Neither Agree not Disagree (3)
- Disagree (2)
- Strongly Disagree (1)
- Don't Know (6)

Please feel free to add additional comments below:

________________________________________________________________

| Page Break |  |
| --- | --- |

Q6 **6) *‘Some of the criteria*** *in the statements above must be fulfilled for a risk factor to be considered truly modifiable: Measurability, potential for change, modifiable cause(s), plausibility, and empirical evidence of effect.’*

- Strongly Agree (5)
- Agree (4)
- Neither Agree nor Disagree (3)
- Disagree (2)
- Strongly Disagree (1)
- Don't know (7)

Please feel free to add additional comments below:

________________________________________________________________

| Page Break |  |
| --- | --- |

Q7 **7)** *‘Some risk factors that were traditionally considered as ‘non-modifiable’, could be considered ‘modifiable’ under certain contexts'* Examples: **Genetics:** the role of **epigenetics.** For example, nutritional and lifestyle interventions leading to changes in oncogenic gene expression in prostate cancer. **Chronic health conditions: remission of Type 2 Diabetes Mellitus** with weight management. **Age:** the **age at which a woman becomes pregnant** may be considered modifiable. **Race and ethnicity:** can be seen as a proxy for other modifiable risk factors. For example, genetics/vitamin D/deprivation/housing as risk factors for **COVID-19 mortality**

- Strongly Agree (5)
- Agree (4)
- Neither Agree nor Disagree (3)
- Disagree (2)
- Strongly Disagree (1)
- Don't Know (6)

Please feel free to add additional comments below:

________________________________________________________________

| Page Break |  |
| --- | --- |

Q8 **8)** *‘Some risk factors should only be considered as* ***partially*** *modifiable i.e. the health risk posed by them can never be completely eliminated’*

- Strongly Agree (5)
- Agree (4)
- Neither Agree nor Disagree (3)
- Disagree (2)
- Strongly Disagree (1)
- Don't know (6)

Please feel free to add additional comments below:

________________________________________________________________

| Page Break |  |
| --- | --- |

Q9 **9)** *‘The extent to which a risk factor is modifiable depends on the disciplinary, social, economic, political, commercial and environmental* ***context****it is used in.’ Examples: Some discipline might classify age, race, and chronic health conditions as*  ***‘non-modifiable’****. However, in certain contexts other disciplines might argue that age as a risk indicator for health can be modified - for example, the age at which women*  ***become pregnant****. In other contexts, age may be closely related to frailty but independent from it, and therefore may not be considered modifiable*

- Strongly Agree (5)
- Agree (4)
- Neither Agree nor Disagree (3)
- Disagree (2)
- Strongly Disagree (1)
- Don't Know (6)

Please feel free to add additional comments below:

________________________________________________________________

End of Block: Please indicate the extent to which you agree with the following statements:

Start of Block: Block 2

Please feel free provide any further comments regarding the use of the term ‘modifiable risk factor’ in your discipline or elsewhere.

________________________________________________________________

End of Block: Block 2

Start of Block: Block 3

Do you think achieving consensus on the definition of modifiable risk factor is important?

- Yes (1)
- No (2)
- Not sure (3)

Please feel free to add additional comments below:

________________________________________________________________

End of Block: Block 3

Start of Block: Introduction

**Supplementary Materials 4: Qualtrics Round 2 Survey**

Thank you for taking part in Round 1 of the DELPHI study that follows on from the opinion piece: ‘Risk factors for ill health: How do we specify what is ‘modifiable’? (Alwan et al.) which recommended establishing consensus on the definition of modifiable determinants of health. Your input for Round 1 was greatly appreciated, and we have taken answers and comments onboard when developing Round 2 of the Delphi. Round 1 Results  Based on the feedback from Round 1, we have modified all statements (including the ones that achieved consensus) to replace the term 'risk factors' with 'health determinants'. These are the four statements that achieved consensus in Round 1 (70% or higher): A modifiable health determinant must be potentially changeable through direct and/or indirect interventions at the individual or population levels (97% agreement) Some of the criteria in the statements above must be fulfilled for a health determinant to be considered truly modifiable: Measurability, potential for change, modifiable cause(s), plausibility, and empirical evidence of effect (82% agreement) Some health determinants that were previously seen as ‘non-modifiable’, could be considered ‘modifiable’ under certain contexts (72% agreement) The extent to which a health determinant is modifiable depends on the disciplinary context and the social, economic, political, commercial and environmental context it is used in (78% agreement) We additionally dropped the following statement given it was mainly covered by the other main statements: ‘Some health determinants should only be considered as partially modifiable i.e. the health risk posed by them can never be completely eliminated’. Round 2  There were a number of statements from Round 1 where consensus was not reached, and following the comments from Round 1, we have adapted these statements accordingly for Round 2.  **The survey will take approximately 5 minutes to complete.**

| Page Break |  |
| --- | --- |

End of Block: Introduction

Start of Block: Demographics

Please state your professional title and name

________________________________________________________________

End of Block: Demographics

Start of Block: Please indicate the extent to which you agree with the following statements:

**For a health determinant to be considered modifiable it must be possible to quantify or describe in some way any potential change in it.**

- Strongly Agree (5)
- Agree (4)
- Neither Agree nor Disagree (3)
- Disagree (2)
- Strongly Disagree (1)
- Don't Know (6)

Please feel free to add additional comments below:

________________________________________________________________

| Page Break |  |
| --- | --- |

**The causes of the modifiable health determinants do not necessarily need to be modifiable in themselves.**

- Strongly Agree (4)
- Agree (6)
- Neither Agree nor Disagree (3)
- Disagree (2)
- Strongly Disagree (1)
- Don't Know (5)

Please feel free to add additional comments below:

________________________________________________________________

| Page Break |  |
| --- | --- |

**For a health determinant to be considered modifiable there needs to be empirical evidence and/or theoretical understanding about its contribution to the causation of the outcome.**

- Strongly Agree (5)
- Agree (4)
- Neither Agree nor Disagree (9)
- Disagree (8)
- Strongly Disagree (7)
- Don't know (6)

Please feel free to add additional comments below:

________________________________________________________________

| Page Break |  |
| --- | --- |

| Page Break |  |
| --- | --- |

**A consensus was reached in Round 1 that 'some of the criteria in the statements must be fulfilled for a health determinant to be considered truly modifiable.' Therefore in Round 2 we would like you to please rank the statements in order of importance with 1 being the most important and 4 the least important. *Please change the ranking of the statements by clicking on them and dragging them up or down.***

______ For a health determinant to be considered modifiable it must be possible to quantify or describe in some way any potential change in it (1)

______ A modifiable health determinant must be potentially changeable through direct and/or indirect interventions at the individual or population levels (2)

______ The causes of the modifiable health determinants do not necessarily need to be modifiable in themselves (3)

______ For a health determinant to be considered modifiable there needs to be empirical evidence or/and theoretical understanding about its contribution to the causation of the outcome (4)

Please feel free to add additional comments below:

________________________________________________________________

End of Block: Please indicate the extent to which you agree with the following statements:

Start of Block: Block 2

Please feel free provide any further comments regarding the use of the term ‘modifiable health determinant’ in your discipline or elsewhere.

________________________________________________________________

End of Block: Block 2
